# Characteristics of Early Mother–Infant and Father–Infant Interactions: A Comparison between Assisted Reproductive Technology and Spontaneous Conceiving Parents

**DOI:** 10.3390/ijerph17218215

**Published:** 2020-11-06

**Authors:** Francesca Agostini, Federica Andrei, Erica Neri, Elena Trombini, Francesca Nuccini, Maria Teresa Villani, Lorenzo Aguzzoli, Marcella Paterlini

**Affiliations:** 1 Department of Psychology, University of Bologna, 40127 Bologna, Italy; federica.andrei2@unibo.it (F.A.); erica.neri4@unibo.it (E.N.); elena.trombini@unibo.it (E.T.); 2 Department of Neonatology, Arcispedale Santa Maria Nuova, Azienda Unità Sanitaria Locale-IRCCS, 42123 Reggio Emilia, Italy; nuccini.francesca@ausl.re.it; 3 Center of Reproductive Medicine and Surgery, Arcispedale Santa Maria Nuova, Azienda Unità Sanitaria Locale-IRCCS, 42123 Reggio Emilia, Italy; MariaTeresa.Villani2@ausl.re.it (M.T.V.); lorenzo.aguzzoli@ausl.re.it (L.A.); 4 Department of Obstetrics and Pediatrics, Arcispedale Santa Maria Nuova, Azienda Unità Sanitaria Locale-IRCCS, 42123 Reggio Emilia, Italy; marcella.paterlini@ausl.re.it

**Keywords:** assisted reproductive technology, parenting, mothers, fathers, parent–infant interactions, parental sensitivity, previous ART, IVF, ICSI

## Abstract

This study aims to describe parents’ and infant’s interactive styles after assisted reproduction treatments (ART), to compare them with parent–infant interactions after spontaneous conception (SC), and to assess the effect of specific ART variables (cause of infertility, treatment type, and previous ART attempts) on interaction quality. The sample included 25 ART conceiving couples and 31 SC couples with their 3-months-old babies. Free parent–infant interactions (3–5 min) were coded using the CARE-Index, a video-based assessment scale that gives both dimensional (e.g., sensitivity, control, passivity) and categorical scores (sensitive, inept, at-risk) for parents and infants. Results showed a global similarity between groups in CARE-Index dimensions. Nevertheless, differences emerged in categorical scores, as the interactive patterns of ART parents were more frequently classified as “inept” and “at-risk” compared to SC parents. With regards to ART dyads only, infants conceived through intracytoplasmic sperm injection scored significantly lower to the dimension compulsivity and higher to passivity, compared to infants conceived through in vitro fertilization. Yet, infants conceived at the first ART cycle had significantly lower levels of difficulty than infants conceived after one ART attempt. These results speak about the existence of important parent–infant interactive differences related to conception modality and ART technique and suggest the need to implement support programs to promote more sensitive parenting styles.

## 1. Introduction

### 1.1. Infertility, Assisted Reproductive Techniques, and Transition to Parenthood

The burden of infertility for couples has become a public health issue in many countries across the world. WHO reported that, in 2010, an estimated 48.5 million couples worldwide were unable to have a child after five years of unprotected intercourse [1]. In Britain, Datta et al. [2] estimated a proportion of 12.5% among women and 10.1% among men who tried unsuccessfully for 12 months or longer to become pregnant. In Italy, infertility is affecting about 15% of couples in childbearing age [3]. Consequently, a high number of couples ask to undergo assisted reproductive techniques (ART), in the hope to achieve the desired pregnancy and baby. Among the most common ART treatments, couples often undergo in vitro fertilization (IVF) technique: women receive hormone injections to stimulate the production of multiple eggs, then the eggs and the male sperm are incubated together in a laboratory dish to produce an embryo, which will later be implanted into the woman’s uterus. In some cases, especially to overcome male fertility problems, intracytoplasmic sperm injection (ICSI) is used: similar to IVF, this technique differs for the fact that it includes a direct injection of a single sperm into each egg to hopefully achieve fertilization.

The scientific community is dedicating a lot of research efforts to investigate medical, biological, environmental, and psychosocial factors associated with both infertility and ART treatments outcomes. Within the psychological domain, attention has been paid to the transition to parenthood in ART couples, however, the investigation still seems at first stages [4,5].

The passage to parenthood is a moment of major life transition, involving relevant changes for both parents from a psychological, sociocultural, and biological perspective [6,7,8]. More strictly, this transition covers the perinatal period, going from conception to the first year after childbirth, reflecting the time for the arrival of the baby and parents’ adjustment. This delicate period is characterized by a certain degree of psychological stress that, according to several variables (e.g., dyadic adjustment, life events, sources of social support, etc.,) may exacerbate into perinatal depression and anxiety [9,10,11,12].

In the context of pregnancies achieved through ART, several findings underline how parental couples may be affected by high levels of psychophysical stress, deriving from multiple factors: the burden of infertility, the duration and type of ART treatments among the first. Infertility related-stress may in fact persist during pregnancy, because of the cumulative negative effect of infertility and ART [4]. Empirical evidence supports a tendency in ART mothers, compared to spontaneously conceiving women, to express more worries, more general and specific anxieties, higher depressive symptoms during the perinatal period [13,14,15,16,17,18,19]. Besides, some studies put in evidence that symptomatology is influenced by the individual history of ART cycles, as women who receive multiple unsuccessful ART treatments would express higher levels of symptomatology [20,21,22].

Hammarberg et al. [4], analyzing different psychological dimensions related to ART parenthood, stated that ART couples may be more frequently characterized by early parenting difficulties, possibly because of a more idealized parenthood and a parental role more complex to achieve. This has been emphasized also by Ranjbar et al. [19], suggesting that the transition to parenthood in ART pregnancies can be psychologically challenging, as the couple moves *“from having an ‘infertile identity’ to a ‘parental identity’”* (p. 2).

In light of the foregoing analysis, it seems relevant to better understand whether the potential difficulties experienced by ART couples during the transition to parenthood may influence early parenting and therefore our attention has been oriented at investigating parent–infant interactions.

### 1.2. Studies on Parent–Infant Interactions in ART Samples

Research on early mother–infant interactions has produced a lot of empirical evidence on the fundamental role they play for the support and promotion of child’s development [23].

Notwithstanding this and considering the stressors that ART parents have to face before giving birth to their baby, mother–infant interactions have been poorly investigated in the context of ART, compared to other psychological dimensions such as depression, anxiety, and parenting stress. Besides, when the research has shown an interest toward the parent–infant relationship after ART pregnancies, studies focusing on the description of interactive behaviours by the use of observative tools have been very few. As Hammarberg et al. [4] emphasized, more generally early parenting after ART treatments and parent–infant relationship still rely on few studies and the ones specifically focused on interactions are even less.

In a study by McMahon et al. [24], 65 mothers who conceived by IVF, compared to 62 control group mothers, rated their baby at 4 months postpartum as more temperamentally difficult and their perception was supported by the interactions observed during the Still Face procedure [25]. In fact, ART infants showed to be fussier, especially for the subgroup of women undergoing repeated ART cycles, while no differences emerged for maternal interactive behaviors. In his review on the development of IVF children, Van Balen [26] concluded that they might be possibly subject to overprotection as they are seen by their parents as very “precious.” Indeed, Gibson et al. [27] found that 65 IVF mothers, at 12 months postpartum, tended to perceive their infants as “special” and more vulnerable; also, when the same researchers [13] assessed the interactive styles in 65 IVF and 61 control group mothers, using the Emotional Availability Scales [28], they found that both groups of mothers were, on average, moderately to generally sensitive toward their infants. Besides, IVF and control infants were rated moderately responsive toward their mothers and engaging them in play. These results were further confirmed by a subsequent study [29], where the Still Face procedure was administered to 70 IVF mothers and 63 controls at 4 months postpartum: significant differences emerged only in terms of more fussiness in IVF infants (during the stressful episode) compared to control ones; other infant measures and maternal sensitivity were similar.

Other studies seem to report different results. Papaliguora and Trevarthen [30] analyzed the characteristics of a 3-min free-play interaction in three groups of mother–infant dyads, based on different methods of conceiving: IVF, standard infertility treatment (INF), and naturally conceiving (SC). Using a specific microanalytic coding system, ART mothers showed a significantly higher “caretaking” behavior, going from 4, 7, 13, to 21 weeks, while it significantly decreased in SC mothers by 21 weeks. Besides, “play” infant behaviors resulted more frequent in IVF and INF infants compared to SC ones. Anyway, because of the limited sample size (8 infants for each group), results need to be interpreted carefully.

Tallandini et al. [31] investigated at 3 months postpartum early mother–infant interactions in an ART sample of 41 mothers compared to 53 SC women, using the Nursing Child Assessment Feeding Scale (NCAFS) [32]. They found that ART mothers interacted positively more frequently with their babies compared to SC ones, in terms of higher provision of “social-emotional and cognitive growth fostering.” No differences emerged, instead, for what concerns the infant behaviors: both ART and SC babies showed similar levels of responsiveness to their mothers. Anyway, it is to note that all the ART infants included in the study were preterm babies, therefore the results cannot be extended to ART full-term infants.

Fewer other studies have also payed attention to fathers, while in some cases the focus was more on self-report measures of father’s attachment to the baby [27,33,34], only the study by La Sala et al. [35] specifically analyzed father–infant interactions. The first three studies found that IVF fathers had similar attachment to the baby to control fathers, both pre and postnatally. La Sala et al. [35] investigated the quality of both mother–infant and father–infant interactions at 12 months postpartum, using the Child Adult Relationship Experimental Index (CARE-Index) [36], on an ICSI sample (39 mothers and 39 fathers) and a control group (45 mothers and 45 fathers). The findings showed lower maternal and paternal sensitivity (intended as a less contingent behavior toward the baby) and higher fathers’ controlling behaviors (in the sense of more intrusive) in the ICSI sample, compared to control; besides, infant cooperation with the father resulted lower for the ICSI sample. Cairo et al. [37] conducted an interesting study, including the administration of pre and postnatal versions of the Lausanne Trilogue Play situation (LTP) [38] to 31 ART couples (83.9% ICSI, 17.1% IVF) and reporting findings on parental attachment and family alliance, but they did not show specific measures on dyadic parent–infant interactions and they did not include a control group.

Globally, what arises with evidence from the literature, is that there is a lack and inconsistency of studies aimed at describing the quality of parent–infant interactions in the first postpartum months following ART treatments. The recent literature review by Allan et al. [5], focusing on the psychosocial factors involved in the transition to parenthood, makes explicit some relevant weaknesses of the literature in the field: first, the transition to parenthood may be affected by prolonged waiting for pregnancy, cause of infertility, and high number of previous ART attempts. Indeed, heterogeneous findings may depend on the characteristics of the ART samples (e.g., type of treatments, non-donor or egg-donation), but also on different times of assessment and tools used. Second, ART fathers’ parenting experiences still are neglected in the research [5].

Therefore, in order to improve our understanding of parent-interactions after ART pregnancies, a cross-sectional study was realized to address the following research questions: (1) Are the characteristics of early parent–infant interactions (at 3 months postpartum) different according to conceiving method (ART versus SC) and to parental role (mother versus father)? We aimed to explore both parents and infants’ specific interactive styles; (2) are the interactive behaviors in the ART sample influenced by the following clinical variables: cause of sterility, treatment type and history of previous ART attempts? Regarding the latter, we considered the presence of previous ART attempts as a possible risk-factor for the quality of early interactions, according to previous studies in literature [20,21,24]. Regarding the cause of sterility and type of ART treatment, as literature has not specifically investigated the role of these variables on early interactions, we considered our study aim as explorative.

## 2. Materials and Methods

### 2.1. Study Design and Participants

This was a cross-sectional study that was realized in the years 2007–2008.

Eligible couples were all contacted at the main public Hospital in Reggio Emilia (Italy), when presenting at around 20 weeks’ gestation for a morphological ultrasound scan; this is a routine obstetric exam aimed to assess main clinical parameters (e.g., fetal size, multiple pregnancies, baby’s physical development and anatomy, position of placenta, and volume of amniotic fluid around the baby).

Inclusion criteria for ART and SC couples were: good understanding of the Italian language, absence of any major complications at childbirth (including preterm births), neonatal or maternal severe disease in the perinatal period. Besides, in accordance with the Hospital guidelines for the admission of patients undergoing ART treatments, maternal age lower than 44 years was considered a further inclusion criterion.

Specifically, for the ART group, couples that reached a non-complicated clinical pregnancy after a successful IVF/ICSI cycle using fresh and ejaculated sperm were considered eligible for the study protocol. Conversely, they were excluded in any case of complications during the ART cycle, defined as any deviation from common clinical course of the cycle, such as risk of ovarian hyperstimulation syndrome (OHSS), a poor ovarian response (less than three follicles), oocyte retrieval complication (i.e., peritoneal bleeding, pelvic sepsis, or abscesses).

A total of 100 couples (38 ART and 62 SC) were considered eligible for the study and were asked to participate to the study. Three ART couples declined, while 15 SC families refused to participate. After childbirth, 13 couples (5 ART and 9 SC) dropped out. Further 16 couples (5 ART and 7 SC) were excluded from the study for methodological issues (e.g., data available only for one parent).

So, the final sample regarded 112 parents (56 couples) and their 56 3-months old babies (52.9% boys). Of these, 25 couples achieved a pregnancy after ART treatments, namely 25 women (mean age = 37.33 years, SD = 3.33) and 25 men (mean age = 39.21 years, SD = 6.53) and their healthy babies. Whereas for the SC group, among eligible couples 31 women (mean age = 33.30 years, SD = 4.80), 31 men (mean age = 34.73 years, SD = 4.74), and their babies were included in the study.

The study was conducted in accordance with the Helsinki Declaration. At enrollment, all women and their partners received oral and written information about the study protocol and signed a written informed consent. The study was not submitted to the Ethics Committee of the Hospital because, at the moment of the study realization (year 2007), the submission for this kind of non-interventional study was not mandatory according to the Italian law. The respect of research ethics guidelines was monitored by the coordinator of the study (Head of the Obstetrics and Gyneacology Unit of the Hospital).

### 2.2. Procedure and Measures

A psychologist met the couples at the Hospital and gave information about the aims of the study, asking their willingness to participate. At enrollment, demographic (e.g., age, place of birth, education, civil status) and clinical data (e.g., date of childbirth, type of delivery, baby’s birth weight and gestational age, baby’s sex) were recorded for all subjects through a specific questionnaire. Additional obstetric variables were collected for the ART group (e.g., cause of infertility, type of ART treatment, number of previous ART attempts).

After the childbirth, families were contacted by phone to schedule a visit at 3 months of infant age. The assessment of the interactions took place in a play-room, set up with a set of standard toys at the hospital. Because all the interactions regarded dyadic interactions (mother–infant and father–infant), while one parent interacted with infant, the other one waited in a different room. The order of parent interaction was randomly chosen. According to the manual of the Child Adult Relationship Experimental Index (CARE-Index) [36], the instructions given to parents were to freely play with their infant “as they usually do at home,” for 3 (minimum) to 5 (maximum) minutes. Mother–infant and father–infant interactions were videorecorded and then coded according to CARE-Index. This tool is based on the observation and evaluation of specific interactive behaviors: facial and verbal expressions, position and body contact, affection, turn-taking contingencies, control and choice of activity [36]. Each behavior is coded to obtain continuous scores (range 0–14) on 7 global scales: 3 scales are for the dimensions of parent’s interactive style (sensitivity, control, and unresponsiveness) and 4 scales are for the infant (cooperation, compulsivity, difficulty, and passivity). The higher the parental sensitivity and infant cooperation scores, the more optimal the dyadic interaction is considered. The CARE Index also provides a classification of a sensitivity scale, based on the categorization of the continuous scores: high-risk (0–4), inept (5–6), and sensitive (scores 7–14) [36]. This coding procedure is suitable for infants from birth to 15 months of age and has been already applied to Italian samples [39,40,41].

All videos were evaluated by two coders, trained and certified as reliable in CARE-Index coding and blind to conceiving method (Cohen’s k = 0.81).

### 2.3. Statistical Analyses

To test for the homogeneity of the two groups, demographic and clinical data were compared between parents who conceived through ART and parents who conceived naturally, using Pearson’s χ^2^ test and Student’s *t* test for independent samples for nominal and continuous variables, respectively.

To explore our first aim, two multivariate analyses of variance-MANOVAs (Pillai’s trace) were conducted. Parents (sensitivity, control, and unresponsiveness) and infants (cooperation, compulsivity, difficulty, and passivity) CARE-Index dimensions were used as outcomes. Each model included conception modality (i.e., ART vs. SC) and parental role (i.e., mother vs. father) as between-subject variables. Additionally, three-way Loglinear analyses were run to investigate for distribution across sensitivity categories (sensitive, inept, and at risk) depending on the conception modality and the parental role.

To explore our second aim, MANOVAs (Pillai’s trace) were further employed. In all models, we explored differences in infants’ and parents’ interactive styles by using parental role (mother vs. father) as between-subject factor, together with either cause of infertility (female factor, male factor, mixed factor), treatment type (IVF vs. ICSI) or the number of previous unsuccessful ART attempts (none, one attempt, more than one attempt).

All statistical analyses were performed using SPSS (version 25) for Windows (IBM, Armonk, NY, USA). In all statistical tests, a *p* value of less than 0.05 was considered significant.

## 3. Results

### 3.1. Demographic and Clinical Characteristics of the Sample

Differences in demographic and clinical variables between ART and SC parents are shown in Table 1.

Overall, all parents were employed and married and 92% of them were born in Italy. Most of them were primiparous (92% and 64.5% of ART and SC couples, respectively). The only statistically significant sociodemographic difference between groups was in age, as mothers and fathers who conceived through ART were older compared to their SC counterparts (F (1, 108) = 7.50, *p* < 0.001). With regards to clinical variables, ART mothers had a significant higher frequency of caesarian section (*χ*^2^ = 6.52, *p*  =  0.011) and ART babies a lower weight at birth (*t* = 2.12, *p*  = 0 .039), compared to SC sample. All these significant differences were somehow expected, because of the nature of ART samples.

Table 2 shows the main data regarding infertility history of ART couples. The prevalent cause of infertility was due to a female factor (44%), mainly endometriosis (55%), followed by a male factor (40%). Couples achieving pregnancy after more than one ART attempt were predominant (40%).

### 3.2. CARE-Index Interactive Styles between ART and SC Dyads

#### 3.2.1. Parents’ Interactive Behaviors

A comparison between ART and SC parents for each of the four CARE-Index parental is shown in Table 3.

Results of a MANOVA, which included conception modality (ART vs. SC) and parental role (mother vs. father) as between-subject variables, showed no significant main (*F* (3, 106) = 2.03, *p* = 0.114, for conception modality), nor interaction (parental role X conception modality; *F* (3, 106) = 0.34, *p* = 0.796) effects for all the CARE-Index dimensions: sensitivity, control, and unresponsiveness.

In light of the results on demographic and clinical characteristics, analyses were rerun by considering the effects of those variables that were significantly different between ART and SC parents and infants. Thus, three separate MANOVAs were retested by controlling for parents’ age, infant birth weight and type of delivery. Neither parents’ age (*F* (3, 106) = 1.48, *p* = 0.223), nor infant birth weight (*F* (3, 106) = 0.13, *p* = 0.939), or type of delivery (*F* (3, 106) = 0.47, *p* = 0.704) added a significant contribution to the results which remained unchanged. Last, we tested a model with infants’ sex as a between-subject factor, but no significant differences emerged (*F* (3, 106) = 0.18, *p* = 0.908).

A three-way Loglinear analysis was performed to further explore the differences in sensitivity range (sensitive, inept, and at risk) by considering conception modality and parental role. While no 3-way interaction emerged, findings revealed only a significant 2-way interaction, specifically between conception modality and sensitivity categories (*χ*^2^ = 7.80, *p*  =  0.020; Z = 2.54, *p* = 0.011): a higher number of SC parents fell into the sensitive category compared to ART parents, who mostly fell in the inept and at risk groups (Table 4).

#### 3.2.2. Infant’s Interactive Behaviors

A comparison between ART and SC parents for each infants’ interactive dimension as assessed by the CARE-Index is shown in Table 5. Results of the MANOVA, which included conception modality and parental role as between-subject variables, showed no significant main (*F* (3, 106) = 2.60, *p* = 0.054, for conception modality), nor interaction (parental role X conception modality; *F* (3, 106) = 0.36, *p* = 0.780) effects for the 4 CARE-Index dimensions: cooperation, compulsivity, difficulty, and passivity.

As in the case for parents’ dimensions of the CARE-Index, three separate MANOVAs were rerun by taking into account the effects of clinical and demographic variables that were significantly different between ART and SC parents and infants. Similarly, neither parents’ age (*F* (3, 106) = 1.86, *p* = 0.140), nor infant birth weight (*F* (3, 106) = 0.81, *p* = 0.492), nor type of delivery (*F* (3, 106) = 0.63, *p* = 0.596), or infants’ sex (*F* (3, 106) = 0.66, *p* = 0.576) added a significant contribution to the results which remained unchanged.

### 3.3. CARE-Index Interactive Styles within the Sample of ART Parents

A number of MANOVAs was rerun to investigate parents’ and infant’s interactive styles within the ART sample, in order to answer the second aim of this study, specifically whether clinical variables related to ART (cause of sterility, treatment type and previous ART attempts) influenced the interactive behaviors in the ART sample. Each model included as between subject factors parental role (mother vs. father) together with one among the following variables: *infertility cause* (female factor, male factor, mixed factors), *treatment type* (IVF vs. ICSI), and *number of previous ART attempts* (none, one, more than one). Means and standard deviations for each of these models are presented in Table 6 (for parents’ CARE-Index dimensions) and Table 7 (infants’ CARE-Index dimensions).

When the variable *infertility cause* was considered, the infertility diagnosis due to unknown factors was not included in the model as group size comprised only one couple (*n* = 1). No main nor interaction effects were found over parents’ CARE-Index dimensions (*F* (4, 84) = 0.85, *p* = 0.499, for the variable cause of infertility; *F* (2, 41) = 0.95, *p* = 0.394, for the variable parental role; *F* (4, 84) = 1.17, *p* = 0.329, for the interaction cause of infertility X parental role); neither over infants’ dimensions (*F* (6, 80) = 0.98, *p* = 0.442, for cause of infertility; *F* (3, 39) = 0.17, *p* = 0.925, for parental role; *F* (6, 80) = 0.65, *p* = 0.692, for cause of infertility X parental role).

When the variable *treatment type* was taken into account, no main nor interaction effects reached statistical significance for parents CARE-Index dimensions (*F* (2, 45) = 2.43, *p* = 0.100, for the variable treatment type; *F* (2, 45) = 2, *p* = 0.146, for the variable parental role; *F* (2, 45) = 0.88, *p* = 0.421, for treatment type X parental role). With respect to infants’ CARE-Index dimensions, a significant main effect for the variable treatment type was found (*F* (3, 43) = 4.83, *p* = 0.005): between subject tests revealed that infants who were conceived through ICSI scored significantly lower to the dimension compulsivity (*F* (1, 45) = 11.70, *p* = 0.001), and higher to passivity (*F* (1, 45) = 7.40, *p* = 0.009) compared to infants conceived through IVF (see Table 7). The main effect of parental role (*F* (3, 43) = 0.59, *p* = 0.625) and the interaction treatment type X parental role (*F* (3, 43) = 0.64, *p* = 0.594) were non-significant.

Regarding *previous ART attempts*, no main nor interaction effects were found over parents’ CARE-Index dimensions (*F* (4, 88) = 0.64, *p* = 0.629, for previous ART attempts; *F* (2, 43) = 1.46, *p* = 0.243, for parental role; *F* (4, 88) = 0.67, *p* = 0.611, for previous ART attempts X parental role). When infants’ CARE-Index dimensions were considered, a significant main effect of previous ART attempts emerged (*F* (6, 84) = 2.60, *p* = 0.023): between subject tests on the outcome variables revealed a significant effect on the dimension difficulty only (*F* (2, 43) = 6.76, *p* = 0.003), and Bonferroni post hoc tests showed that infants who were conceived at the first ART attempt had significantly lower levels of difficulty (*M* = 2.30, *SD* = 1.70), compared to infants conceived after one ART attempt (*M* = 4.43, *SD* = 1.82; *p* = 0.003) (see Table 7).

No significant differences emerged between the group of infants conceived after more than one attempt and the other two groups (*M* = 3.30, *SD* = 1.41; *p* = 0.137 and *p* = 0.156 when compared with the groups conceived after no previous ART attempts and one ART attempt, respectively). The main effect of parental role (*F* (3, 41) = 0.35, *p* = 0.782) and the interaction previous ART attempts X parental role (*F* (6, 84) = 0.69, *p* = 0.653) were non-significant.

## 4. Discussion

The assessment of the quality of early interactions represents a relevant topic in the research on the transition to parenthood. Particularly, the presence of sensitive interactive styles in parents is associated with an appropriate child development. However, stressful experiences (i.e., infertility and assisted reproductive treatments) during the passage to parenthood may influence mothers’ and fathers’ mental health and this, in turn, may impact on their early parenting and interactive abilities. Nevertheless, to our knowledge, only few studies investigated the effect of ART on parent–infant interactions, leading to heterogeneous findings. Giving the relevance of this topic, the aim of the study was to deepen the knowledge on the quality of early interactions in ART parent–infant dyads.

Globally, the main differences in the quality of early interactions between ART and SC parents regarded sensitivity; besides, within the ART sample, type of treatment and previous ART attempts influenced differently infants’ interactive behaviors.

Our first aim was to compare parental interactive patterns according to the conceiving method (ART versus SC). All mothers’ and fathers’ interactive behaviors were similar between the two groups and this resulted in line with previous literature, where ART parents at 4 months postpartum were described sensitive as SC couples when interacting with their infants [24,29]. However, two further studies [30,31] found better outcomes in ART parents than in control group, up to 3–5 months postpartum, while La Sala et al. [35] observed a higher intrusiveness in the ART sample.

Such mixed results could be explained by methodological issues. First, previous studies greatly vary according to the time of assessment: some of them focused on 3–5 months [29,30,31,34] or 12 months of life [13,29,35]. These periods of infant’s age represent significant and distinct steps for the co-construction of dyadic interactive patterns and infant development [23,42], so it is reasonable to obtain different results, as an expression of different infant skills and abilities. According to our study, we specifically chose to observe the 3 month-period because it is a relatively stable developmental stage, where the baby starts to become more socially active, alert, and communicative and shows an increasing ability for *face-to-face* interactions.

Another methodological issue concerns the characteristics of the instrument chosen to assess early interactions. The use of a wide range of observative scales in previous studies [13,24,30,31,43] reflects the operationalization of different constructs to describe interactive behaviors and some variables could not correspond or only partially overlap. This aspect could contribute to contrasting findings. In the present study, we chose to use the CARE-Index [36] because it is an observational scale focused on the construct of parental sensitivity and able to detect non optimal patterns, such as intrusive or withdrawal dimensions (control and unresponsiveness scales, respectively).

Beyond the dimensional evaluation of early interactions, another feature of CARE-Index is the classification of the level of parental sensitivity, that allows to differentiate “good enough” interactions [44], from less sensitive ones: those with more severe moments of mismatch and limited playfulness (inept), and those with evident parental hostility and clear absence of empathy (high-risk). According to Crittenden [36], these two categories are clinically relevant because they facilitate the identification of at-risk parenting contexts and give direction for the need of psychoeducational intervention or psychotherapy. This classification becomes quite significant in the present study: while no differences emerged with respect to CARE-Index dimensions, an evident effect of the conceiving method was observed for the level of sensitivity, as ART parents showed more frequently inept and at-risk interactive styles.

Taken together, these findings on CARE-Index outcomes suggest that the investigation of the quality of dyadic interactions requires the use of very accurate instruments, in order to increase the chance to detect existing differences related to our study variables (in this case conceiving method). In this study, using both CARE-Index types of evaluation (dimensional and categorical), it was possible to explore two different aspects of parental sensitivity. Also Wang et al. [45], in a meta-analysis on parenting styles after IVF treatments, showed that the influence of ART may be expressed on a wide range of maternal dimensions (e.g., warmth, controlling, rejection), according to specific characteristics of the samples. This would confirm the benefit of using precise and appropriate observational scales.

Another key aspect of the study is the inclusion of both mothers and fathers, as we aimed to explore whether parental gender could interact with conceiving method in influencing the quality of interactions. This aim is supported by the evidence of a growing interest on the investigation of the paternal role in the last years [46,47,48], also in well-known risk conditions for parenting [40,49,50,51].

Despite the literature underlines that early father–infant interactions may differ from mother–infant ones, showing more animated and less sensitive patterns in the first ones [52,53,54,55,56], our findings showed similar interactive behaviors between mothers and fathers, independently from conceiving method. We may hypothesize that the higher involvement that ART treatments require to both women and men persist also after birth, enhancing the synergy inside the parental couple, leading the couple to be grateful and both mother and father to a very active engagement in the care of the infant [13,33,57]. Indeed, as underlined by Cohen et al. [33] and Hjelmstedt and Collins [34], ART fathers showed a level of attachment and satisfaction of their parental role very similar to those observed in ART mothers and to control fathers. Regarding SC couples, results showed quite similar interactive behaviors on all CARE-Index dimensions and this may reflect a growing tendency, confirmed by recent empirical evidence, to develop co-parenting shared patterns and to exert mutual parental influence on early parenting styles [58,59]. Besides, as only recent literature is showing interest in paternal interactive behaviors, previous studies have frequently investigated fathers’ early interactive behaviors with instruments originally developed for mother–infant interactions. Conversely, in the present study the use of CARE Index for both mothers and fathers was supported by recent literature investigating paternal interactive styles [36,40,60,61].

According to our first aim, we did not find a significant influence of the conceiving method on infant interactive patterns. This result is consistent with previous studies [13,27,31], but not with others [24,29,35], where ART infants where described as fussier and less cooperative, when compared to control group.

Again, we should consider the influence of some methodological factors. In the studies by McMahon et al. [24] and McMahon and Gibson [29], ART samples included couples undergoing only IVF treatments, while our sample included IVF and ICSI subjects. Besides, in studies [24,29], infant fussiness, detected at approximately the same age of our study (4 months), was detectable only during the Still-Face segment of the Still-Face procedure. No differences emerged during the other phases, where spontaneous and play interaction usually take place; this is similar to what we observed during the free parent–infant interactions in ART and SC infants of our study. All these findings could suggest that ART infants may be particularly sensitive to the adaptation to stressful situations. Regarding the study by La Sala et al. [35], the only one that assessed dyadic interactions by CARE Index, we may speculate that the different results (the authors observed less cooperative behaviors in ART infants) are not inconsistent and are partially related to the infant’s age considered (12 months): the specific interactive configurations change largely during the first year of life, therefore it may be possible that the findings reflect the developmental trajectories of infant interactive behaviors. Anyway, to our knowledge, interactions in a longitudinal perspective have never been explored in ART infants, so a future investigation would be beneficial.

The last aim of the study was to explore the influence of clinical factors (infertility cause, treatment type and number of previous ART attempts) on interactive patterns of ART dyads. Considering that ART treatments are quite heterogeneous in terms of type, duration, cycles, etc., it is relevant to consider the possible influence of these factors on the investigated variables, especially within the psychological domain, as previous literature showed a lack in this kind of investigation.

Overall, no significant differences emerged for parents’ interactive behaviors with relation to all ART variables, nor for infant patterns considering infertility cause. Conversely, infant interactive behaviors resulted different depending on the treatment type and to the number of previous ART attempts. Before going into details with comments, we have to keep in mind that, due to the small size of ART sample, these findings should be considered preliminary.

When the effect of treatment type was considered, lower compulsive and higher passive interactive styles differentiated IVF infants from ICSI group. According to CARE-Index definition, both compulsivity and passivity represent not optimal patterns, anyway the first one is somehow more evolved, because it requires the infant to inhibit its reaction to parent stimulations [36]; conversely, passivity implies self-absorption and withdrawal, it occurs in younger infants and especially in response to remote or depressed parents [36]. In the hospital where dyads were recruited, couples underwent ICSI treatment only in case of infertility with high clinical severity given by male factors or repeated failures in IVF. Such condition may have affected the quality of parent–infant interactions, whose effects have been expressed by infants who were significantly more passive than their IVF counterparts and by higher levels of unresponsiveness for ICSI compared to IVF parents, although non-significant. Further studies with larger IVF and ICSI samples should deepen this issue; to our knowledge, in fact, only two previous studies investigated the influence of treatment type on parent-child interactions [43,62], but considering different modalities of treatment (egg donation and IVF) and child’s age (6–18 months, and 2–5 years, respectively). Besides, these two studies did not include a control group, therefore overall comparisons are difficult to make.

Regarding previous ART attempts, infants who were conceived after one previous ART treatment showed more difficult interactive behaviors than those conceived at first cycle. A similar result emerged in a previous study where, even if using a different observational procedure [24], the authors observed infant fussiness significantly more frequently in the case of previous ART repeated cycles. As other studies underlined, a higher number of previous ART attempts may negatively influence parents’ mental health [21,22], therefore this clinical variable could play a role also with regards to the quality of early interactions. Anyway, it should be noted that this effect emerged for infants conceived after one previous ART attempt, but not for infants conceived after more than one. There is the need to further replicate the study with wider samples, in order to possibly confirm these findings. At the same time, it is important to keep in mind that some ART parents, after more than one attempt failure, would be more keen on developing a high level of resilience, supported by their very strong desire for parenthood [63], and this could act as a protective factor for infant mental health.

Globally, these results regarding the impact of the ART clinical variables add new knowledge to the literature about infants’ early interactive behaviors, poorly investigated until now in ART samples. While few previous studies underlined a negative effect especially in terms of higher irritability and emotional dysregulation [24,29,35], we also found a tendency to withdrawal and passivity, that requires further research. Taken together, this study suggests that ART may exert varied and multiple effects on early interactions.

### Limitations and Strengths

Some limitations of the study should be remarked. First, the sample size could be enhanced in order to allow more sophisticated analyses. This is particularly relevant when the influence of ART clinical factors (infertility cause, treatment type, and number of previous ART attempts) was considered. Regarding this, we also highlight that our sample did not include cases of donor fertilization, because at the time of the realization of the study (year 2007–2008) this was not allowed by Italian law. Second, the influence of other factors, both demographic (like number of previous children per couple and actual infants’ age), as well as emotional (such as parents’ depressive and anxiety symptoms), was not considered. As infertility may be associated with an increased risk of depression and anxiety [14,15,16,17,22] and affective symptomatology may impair the quality of early interactions [64,65], further studies should investigate whether the differences in maternal and paternal interactive styles in ART samples are influenced by their affective states. Finally, future studies on the same topic study would benefit from a longitudinal design, aimed at understanding the trajectories of the interactive patterns of ART parent–infant dyads. All these aspects need to be considered for their impact on the internal validity of the study and on the possibility to extend the generalizability of our findings (external validity).

Notwithstanding the above limitations, several strengths should also be mentioned. Despite research interest was paid to investigate parenting and parent–infant relationship in ART samples, only few studies accurately investigated early interactions. Indeed, studies often focused on wide range of definitions of dyadic interaction, assessing constructs like parent–infant relationship, pre and postnatal attachment or parental adjustment, by using self-report measures. Therefore, a strength of our study is the use of an observational scale specifically developed to describe interactive patterns. The CARE-Index is also suitable for the potential clinical relevance of its dimensions. The identification of dyads at risk, based on the lack in sensitivity, can be considered a useful resource to implement ad hoc early interventions, in order to prevent negative consequences on later child’s development.

Besides, another element of strength is represented by the inclusion of specific ART variables and the exploration of their possible role regarding quality of interactions. As ART samples are usually heterogeneous and characterized by many different medical variables, having the possibility to control some of them may increase the possibility to describe with accuracy what is influencing the interactive patterns.

Finally, a strength of this study regards the focus on fathers, a population which the international literature is recently becoming interested in.

## 5. Conclusions

The present study evaluated early interactions in ART parent–infant dyads, through a validated observational scale and by considering the effect of specific ART treatment variables.

In summary, assisted reproductive techniques seem to partially influence the co-construction of parent–infant interactions, as the effects are associated to the clinical conditions around ART pregnancy. Dimensional interactive patterns were similar between ART and SC parents, but particular attention should be paid to a general lack in sensitivity in ART samples, as important cue to detect families in need of intervention. So, an accurate assessment of the quality of early interactions is recommended. Furthermore, no differences between maternal and paternal interactive patterns were observed, suggesting that the effect of ART on the parents seems to globally influence the couple, independently from parental gender, leading them to respond to the demand of the transition to parenthood in a similar way.

Conversely, a negative effect of ART on infant early interactive behaviors appeared to be significant in presence of specific clinical variables, such as ICSI treatment and a previous ART attempt. Both elements may represent additional factors of distress, which contribute to increase the cumulative stress already experienced by ART couples, during the transition to parenthood, because of infertility and ART treatments. This could explain the negative impact on the quality of parenting, with consequences on infants’ interactive behaviors as we observed in the present study.

Overall, the present study adds new findings to the literature and have clinical and practical implications for the improvement of preventive interventions to support parenting in ART couples; specifically, the results highlight the relevance of supporting parent–infant relationship after ART pregnancies in presence of specific clinical risk conditions.

## Figures and Tables

**Table 1:** Demographic and clinical characteristics in assisted reproduction treatments (ART) and spontaneous conception (SC) parents.

	ART (N = 50)			SC (N = 62)			
Sample Characteristics	Fathers (N = 25)	Mothers (N = 25)	Total (N = 50)	Fathers (N = 31)	Mothers (N = 31)	Total (N = 62)	*p*
Parents’ characteristics							
Mean age in years (*SD*)	39.2 (6.53)	37.3 (3.33)	38.3 (5.21)	34.7 (4.73)	33.3 (4.80)	34.02 (4.78)	0.001
Place of birth, *n* (%)							0.500
Italy	23 (46)	23 (46)	46 (92)	29 (46.70)	28 (45.10)	57 (91.90)	
Abroad	2 (4)	2 (4)	4 (8)	2 (3.20)	3 (4.80)	5 (8.10)	
Level of education, *n* (%)							0.650
Secondary school	4 (16)	2 (8)	6 (12)	7 (22.60)	2 (6.50)	9 (14.50)	
High school	11 (44)	14 (56)	25 (50)	15 (48.40)	12 (38.70)	27 (43.50)	
University	10 (40)	9 (36)	19 (38)	9 (29)	17 (54.80)	26 (42)	
Previous deliveries, *n* (%)							0.180
None			23 (92)			20 (64.50)	
One			1 (4)			9 (29)	
More than one			1 (4)			2 (6.50)	
Type of delivery, *n* (%)							
Natural childbirth			10 (40)			24 (77.40)	0.011
Caesarian section			13 (52)			7 (22.60)	
Children’s characteristics							
Mean gestational age at birth in weeks (*SD*)			38.44 (2.90)			39.31 (1.15)	0.130
Mean birth weight in grams (*SD*)			3020 (0.53)			3320 (0.52)	0.039
Sex, *n* (%)							
Male			11 (44)			19 (61.30)	0.197
Female			14 (56)			12 (38.70)	

**Table 2:** Clinical data regarding sub-fertile couples who conceived through ART.

Variable	ART Couples (N = 25)
Cause of infertility, *n* (%)	
Female	11 (44)
Male	10 (40)
Both	3 (12)
Unknown	1 (4)
Treatment type, *n* (%)	
ICSI ^1^	15 (60)
IVF ^2^	10 (40)
Mean number of ART attempts (SD), *range* Previous ART attempts, *n* (%)	1.38 (1.30), 0–4
None	8 (32)
One	7 (28)
More than one	10 (40)

Note. ^1^ ICSI = intracytoplasmic sperm injection, ^2^ IVF = In vitro fertilization.

**Table 3:** Mean ± standard deviation (range) for interactive styles (CARE-Index dimensions) in ART and SC parents.

	ART (N = 50)			SC (N = 62)		
CARE-Index Dimensions	Fathers (N = 25)	Mothers (N = 25)	Total (N = 50)	Fathers (N = 31)	Mothers (N = 31)	Total (N = 62)
Sensitivity	5.60 ± 2.02	5.40 ± 1.73	5.50 ± 1.86	6.35 ± 2.10	6.13 ± 1.94	6.24 ± 2.01
	(3–11)	(3–9)	(3–11)	(2–11)	(2–10)	(2–11)
Control	3.40 ± 2.41	4.68 ± 2.52	4.04 ± 2.53	2.81 ± 2.92	3.77 ± 2.95	3.29 ± 2.95
	(0–9)	(0–10)	(0–10)	(0–9)	(0–10)	(0–10)
Unresponsiveness	5.00 ± 2.94	3.92 ± 2.73	4.46 ± 2.86	4.77 ± 3.11	4.10 ± 2.97	4.44 ± 3.03
	(0–10)	(1–10)	(0–10)	(0–10)	(0–10)	(0–10)

**Table 4:** Distribution of frequencies for sensitivity range between ART and SC parents.

	ART (N = 50)			SC (N = 62)		
Sensitivity Range	Fathers (N = 25)	Mothers (N = 25)	Total (N = 50)	Fathers (N = 31)	Mothers (N = 31)	Total (N = 62)
Sensitive	4 (16)	7 (28)	11 (22)	14 (45.20)	15 (48.40)	29 (46.80)
Inept	16 (64)	8 (32)	24 (48)	12 (38.70)	10 (32.20)	22 (35.50)
At risk	5 (20)	10 (40)	15 (30)	5 (16.10)	6 (19.40)	11 (17.70)

Note. Data are expressed as frequencies (%).

**Table 5:** Mean ± standard deviation (range) for interactive styles (CARE-Index dimensions) in ART and SC infants.

	ART (N = 50)			SC (N = 62)		
CARE-Index Dimensions	Fathers (N = 25)	Mothers (N = 25)	Total (N = 50)	Fathers (N = 31)	Mothers (N = 31)	Total (N = 62)
Cooperation	5.08 ± 2.08	4.92 ± 2.08	5.00 ± 2.06	6.45 ± 2.42	5.71 ± 2.42	6.08 ± 2.43
	(2–10)	(2–10)	(2–10)	(2–12)	(0–10)	(0–12)
Compulsivity	1.63 ± 1.74	2.20 ± 1.89	1.92 ± 1.82	1.65 ± 2.51	2.65 ± 2.27	2.15 ± 2.42
	(0–5)	(0–6)	(0–6)	(0–9)	(0–7)	(0–9)
Difficulty	3.25 ± 1.96	3.36 ± 1.65	3.31 ± 1.79	2.35 ± 1.90	3.13 ± 2.81	2.74 ± 2.41
	(0–7)	(1–6)	(0–7)	(0–9)	(0–14)	(0–14)
Passivity	4.04 ± 2.66	3.52 ± 2.48	3.78 ± 2.56	3.55 ± 2.85	2.52 ± 2.63	3.03 ± 2.77
	(0–10)	(0–9)	(0–10)	(0–10)	(0–9)	(0–10)

**Table 6:** Interactive styles (CARE-Index dimensions) in ART parents by infertility cause, treatment type, and number of previous ART attempts.

	Parental Dimensions								
	Sensitivity			Control			Unresponsiveness		
	Fathers (N = 25)	Mothers (N = 25)	Total (N = 50)	Fathers (N = 25)	Mothers (N = 25)	Total (N = 50)	Fathers (N = 25)	Mothers (N = 25)	Total (N = 50)
Infertility cause									
Male factor	5.50 ± 2.01	6.10 ± 1.72	5.80 ± 1.85	3.60 ± 3.06	3.70 ± 2.40	3.65 ± 2.68	4.90 ± 3.07	4.2 ± 2.97	4.55 ± 2.96
Female factor	5.91 ± 1.86	4.64 ± 1.43	5.27 ± 1.75	3.45 ± 2.25	5.91 ± 2.58	4.68 ± 2.67	4.64 ± 2.76	3.45 ± 2.62	4.05 ± 2.69
Mixed factors	5.67 ± 3.05	6.33 ± 2.08	6.00 ± 2.36	2.67 ± 1.15	3.67 ± 1.52	3.17 ± 1.32	5.67 ± 4.16	4.00 ± 3.46	4.83 ± 3.54
Treatment type									
IVF ^1^	6.00 ± 1.63	5.00 ± 1.70	5.50 ± 1.70	4.00 ± 1.94	5.90 ± 2.76	4.95 ± 2.52	4.00 ±2.86	3.10 ± 2.72	3.55 ± 2.76
ICSI ^2^	5.33 ± 2.25	5.67 ± 1.75	5.50 ± 1.99	3.00 ± 2.67	3.87 ±2.06	3.43 ± 2.38	5.67 ± 2.85	4.47 ±2.69	5.07 ± 2.81
Number of previous ART attempts									
None	5.63 ± 1.50	5.03 ± 1.72	5.38 ± 1.58	3.63 ± 3.15	3.87 ± 2.74	3.75 ± 2.86	4.75 ± 3.61	5.00 ± 3.3	4.88 ± 3.36
One	4.57 ± 0.78	5.43 ± 1.81	5.00 ± 1.41	3.57 ± 1.71	5.29 ± 1.97	4.43 ± 1.98	5.86 ± 1.67	3.29 ± 1.79	4.57 ± 2.13
More than one	6.30 ± 2.71	5.60 ± 1.83	5.95 ± 2.82	3.10 ± 2.37	4.90 ± 2.76	4.00 ± 2.67	4.60 ± 3.20	3.5 ± 2.75	4.05 ± 2.96

Note. ^1^ ICSI = Intracytoplasmic Sperm Injection, ^2^ IVF = In Vitro Fertilization.

**Table 7:** Interactive styles (CARE-Index dimensions) in ART infants by infertility cause, treatment type, and number of previous ART attempts.

	Infant Dimensions											
	Cooperation			Compulsivity			Difficulty			Passivity		
	Fathers (N = 25)	Mothers (N = 25)	Total (N = 50)	Fathers (N = 25)	Mothers (N = 25)	Total (N = 50)	Fathers (N = 25)	Mothers (N = 25)	Total (N = 50)	Fathers (N = 25)	Mothers (N = 25)	Total (N = 50)
Infertility cause												
Male factor	5.10 ± 1.91	5.90 ± 2.07	5.50 ± 1.98	1.30 ± 2.05	1.40 ± 1.64	1.35 ± 1.81	3.10 ± 1.79	3.10 ± 1.91	3.10 ± 1.80	4.50 ± 2.99	3.60 ± 2.91	4.05 ± 2.91
Female factor	5.40 ± 2.06	4.00 ± 16.7	4.67 ± 1.95	2.00 ± 1.63	3.27 ± 1.84	2.67 ± 1.82	3.10 ± 2.18	3.36 ± 1.12	3.24 ± 1.67	3.50 ± 2.55	3.36 ± 1.96	3.44 ± 2.20
Mixed factors	5.00 ± 3.00	5.33 ± 2.88	5.17 ± 2.63	2.00 ± 1.00	1.67 ± 1.15	1.83 ± 0.98	3.00 ± 1.00	3.33 ± 2.51	3.17 ± 1.72	4.00 ± 3.00	3.67 ± 4.04	3.83 ± 3.18
Treatment type												
IVF ^1^	5.56 ± 1.81	4.40 ± 1.71	4.95 ± 1.81	2.56 ± 1.66	3.30 ± 1.70	2.95 ± 1.68 ^3^	3.22 ± 2.27	3.80 ± 1.31	3.53 ± 1.80	2.67 ± 2.29	2.50 ± 2.27	2.68 ± 2.21 ^4^
ICSI ^2^	4.80 ± 2.24	5.27 ± 2.28	5.03 ± 2.23	1.07 ± 1.58	1.47 ± 1.68	1.27 ± 1.61 ^3^	3.27 ± 1.83	3.07 ± 1.80	3.17 ± 1.80	4.87 ± 2.58	4.20 ± 2.45	4.53 ± 2.50 ^4^
Number of previous ART attempts												
None	5.14 ± 1.57	4.50 ± 1.85	4.80 ± 1.69	2.71 ± 2.36	2.63 ± 1.99	2.67 ± 2.09	1.57 ± 0.97	2.88 ± 1.95	2.27 ± 1.66 ^3^	4.57 ± 3.50	4.00 ± 3.33	4.27 ± 3.30
One	4.14 ± 1.21	5.00 ± 2.51	4.57 ± 1.95	1.29 ± 1.11	1.86 ± 1.86	1.57 ± 1.50	4.57 ± 2.22	4.29 ± 1.49	4.43 ± 1.82 ^3^	4.00 ± 2.38	2.86 ± 1.9	3.43 ± 2.17
More than one	5.70 ± 2.71	5.20 ± 2.09	5.45 ± 2.37	1.10 ± 1.37	2.10 ± 1.96	1.60 ± 1.72	3.50 ± 1.50	3.10 ± 1.37	3.30 ± 1.41	3.70 ± 2.40	3.60 ± 2.17	3.65 ± 2.23

Note. ^1^ IVF = in vitro fertilization, ^2^ ICSI = intracytoplasmic sperm injection; ^3^ *p* = 0.001, ^4^ *p* = 0.009.
